# Beta bursts in the parkinsonian cortico-basal ganglia network form spatially discrete ensembles

**DOI:** 10.1016/j.nbd.2024.106652

**Published:** 2024-10-15

**Authors:** Isaac Grennan, Nicolas Mallet, Peter J. Magill, Hayriye Cagnan, Andrew Sharott

**Affiliations:** a Medical Research Council Brain Network Dynamics Unit, Nuffield Deptartment of Clinical Neurosciences, University of Oxford, Oxford OX1 3TH, United Kingdom; b Universite de Bordeaux, Institut des Maladies Neurodégénératives, 33076 Bordeaux, France; c CNRS UMR 5293, Institut des Maladies Neurodégénératives, 33076 Bordeaux, France; d Department of Bioengineering, Sir Michael Uren Hub, Imperial College London, London, W12 0BZ, UK

**Keywords:** Parkinson's disease, beta oscillations, Pathophysiology, Deep brain stimulation, Synchrony

## Abstract

Defining spatial synchronisation of pathological beta oscillations is important, given that many theories linking them to parkinsonian symptoms propose a reduction in the dimensionality of the coding space within and/or across cortico-basal ganglia structures. Such spatial synchronisation could arise from a single process, with widespread entrainment of neurons to the same oscillation. Alternatively, the partially segregated structure of cortico-basal ganglia loops could provide a substrate for multiple ensembles that are independently synchronized at beta frequencies. Addressing this question requires an analytical approach that identifies *groups* of signals with a statistical tendency for beta synchronisation, which is unachievable using standard pairwise measures. Here, we utilized such an approach on multichannel recordings of background unit activity (BUA) in the external globus pallidus (GP) and subthalamic nucleus (STN) in parkinsonian rats. We employed an adapted version of a principle and independent component analysis-based method commonly used to define assemblies of single neurons (i.e., neurons that are synchronized over short timescales). This analysis enabled us to define whether changes in the power of beta oscillations in local ensembles of neurons (i.e., the BUA recorded from single contacts) consistently covaried over time, forming a “beta ensemble”. Multiple beta ensembles were often present in single recordings and could span brain structures. Membership of a beta ensemble predicted significantly higher levels of short latency (<5 ms) synchrony in the raw BUA signal and phase synchronisation with cortical beta oscillations, suggesting that they comprised clusters of neurons that are functionally connected at multiple levels, despite sometimes being non-contiguous in space. Overall, these findings suggest that beta oscillations do not comprise of a single synchronisation process, but rather multiple independent activities that can bind both spatially contiguous and non-contiguous pools of neurons within and across structures. As previously proposed, such ensembles provide a substrate for beta oscillations to constrain the coding space of cortico-basal ganglia circuits.

## Highlights

• Temporally correlated changes in beta power can be used to define “beta ensembles”
• Approach was applied to multichannel basal ganglia recordings in parkinsonian rats.
• Multiple ensembles were present in single recordings and could span brain structures.
• Beta ensembles could bind spatially contiguous and non-contiguous pools of neurons
• Parkinsonian beta oscillations comprise multiple, independent activities.

## 1 Introduction

Beta oscillations (15 to 35 Hz) across the primary motor cortex and basal ganglia are enhanced during Parkinson's disease (PD) (Brown, 2007; Hammond et al., 2007). Effective therapies for PD, such as deep brain stimulation (DBS) and levodopa administration, reduce beta power and the degree of suppression correlates with improvement in bradykinetic symptoms (Brown et al., 2001; Kuhn et al., 2008). Beta oscillations are not stationary, but rather occur in “bursts” that consist of transient increases in the instantaneous power of basal ganglia local field potentials (LFPs) or the electrocorticogram (ECoG) (Feingold et al., 2015; Cagnan et al., 2019). OFF levodopa, beta bursts increase in duration and amplitude (Tinkhauser et al., 2017a). The reduction in the number of beta bursts and suppression of long-duration bursts by treatment (e.g. levodopa or DBS) are correlated with the degree of improvement in motor performance (Little et al., 2013; Tinkhauser et al., 2017a).

A key question in the field of PD pathophysiology is why enhanced beta oscillations lead to motor symptoms. Several authors have proposed that the level of beta synchronisation influences the dimensionality of the coding space in the cortico-basal ganglia circuits (Engel et al., 2001; Brittain et al., 2014). In this framework, individual neurons or small ensembles of neurons should have a relatively large degree of independence to function optimally. This is supported by the low-level of temporal correlation between basal ganglia neurons in awake animals (Raz et al., 2001). Short periods of enhanced synchronisation (<1 s), however, could be utilized to hold a population of neurons in a given state (Engel et al., 2001; Brittain et al., 2014). In the healthy motor system, transient periods of synchronisation could support muscle synergies to hold part of the limb still (Bracklein et al., 2022). However, if these periods of synchronisation become abnormally sustained, they could impair dynamic processing and execution of the associated behaviour.

Beta synchronisation can occur in both temporal and spatial dimensions. Detailed examination of beta bursts has demonstrated that beta oscillations in the parkinsonian brain are pathologically extended in time (Tinkhauser et al., 2017b; Tinkhauser et al., 2017a). The length of the cortical (ECoG) burst predicts duration of cortico-basal phase-locking at the level of single- and multi-units (Cagnan et al., 2019; Baaske et al., 2020) suggesting that the elongation of beta bursts in LFP is representative of oscillation at the level of neuronal output (Cagnan et al., 2019). Given that field signals (EEG/ECoG/LFP) are the result of temporally synchronized synaptic potentials across neurons (Buzsaki et al., 2012), sustained bursts also indicate enhanced spatial synchronisation at a local level around the electrode. However, any spatial synchronisation between different field potentials recorded within or across structures, indicating coupling over larger spatial scales, will be a composite of volume conduction, the reference configuration and physiological coupling. LFP/ECoG-based analyses will thus provide only superficial insights into spatial synchronisation at the level of neuronal populations.

With respect to unit activity, the beta coherence between the spiking of pairs of units in the rodent GP decreases with the distance between the recorded pair (Mallet et al., 2008b). However, neurons in the cortex, basal ganglia and thalamus are clustered into hundreds of semi-independent pathways that may not conform to simple gradients in one or more anatomical plane (Hunnicutt et al., 2016; Foster et al., 2021). Neurons in a given brain structure could divide bimodally into populations that always do or do not engage with the same unified process. Alternatively, there could be independent assemblies of neurons that are synchronized at beta frequencies at different times. To distinguish between these scenarios requires analyses of the spatiotemporal dependence of the synchronisation of neuronal output using a method that does not rely on signals being spatially contiguous and that can detect different ensembles of neurons that are independent of one another.

Here we employed an analytical framework using principal component and independent component analyses, commonly used to define groups of neurons with a tendency to co-fire over short-timescales (i.e. neural ‘ensembles’) (Lopes-dos-Santos et al., 2011; Lopes-dos-Santos et al., 2013; van de Ven et al., 2016). We applied this approach to multichannel background unit activity (BUA) recorded in 6-OHDA-lesioned rats in the external globus pallidus (GP) and subthalamic nucleus (STN) to define features of the spatiotemporal synchronisation of pathological beta oscillations. The results show that multiple, independent ensembles of neurons are coordinated by the temporal dynamics of ongoing beta oscillations, and that neurons comprising these ‘beta ensembles’ can be distributed across brain structures.

## 2 Methods

### 2.1 Parkinsonian rats

This study uses data from Mallet et al., 2008a. Extracellular recordings were made using multichannel silicon probes in the GP and STN of urethane-anaesthetised Sprague-Dawley rats (*n* = 15) rendered parkinsonian by 6-OHDA-lesioning. 6-OHDA lesioning was carried out under anaesthesia by injecting 1 microL of 6-OHDA 1.2–1.4 mm lateral and 4.1 mm posterior to Bregma and 7.9 mm ventral to the dura. Animals that recovered successfully and were deemed to have been effectively lesioned were used for electrophysiological recordings. Lesions were classified as effective if 15 days after lesioning on administration of apomorphine animals performed ≥ 90 contraversive rotations in 20 min. Recordings were made on a pair of probes, each with 16 electrodes arranged in a single vertical plane separated by 100 μm. In some recordings, one probe was in the GP and one in the STN (*n* = 7/45 recordings) whereas, in others, probes made up of two shanks separated by 500 μm were targeted to the GP (*n* = 38/45 recordings). The number of electrodes within targeted structures varied between recordings. Channels outside of the STN and GP were excluded from the analysis. Frontal ECoG recordings, ipsilateral to the lesion, were made simultaneously with those in the basal ganglia using a 1 mm-diameter screw above the frontal motor cortex referenced to a screw overlying the ipsilateral cerebellar hemisphere. Signals were recorded using a Power 1401 amplifier and Spike2 (Cambridge Electronic Design Limited) at a sample rate of 16 kHz. Recording duration was on average 108.1 s +/− 6.7 s. Following recordings animals were euthanised and fixed by transcardial perfusion.

For Supplemental Fig. 5, 8 recordings from unlesioned, control Sprague-Dawley rats (*n* = 5) were used. These animals went through the same recording procedure as parkinsonian rats in the absence of 6-OHDA lesioning. Recordings were again made with a pair of 16 electrode probes, either both targeted to the GP (3/8) or one targeted to the GP and the other to the STN (5/8).

Data presented for the rest of the paper was from parkinsonian animals unless stated otherwise.

### 2.2 Power spectrum and coherence

The power spectrum and coherence were calculated as in Cagnan et al., 2019. ECoG and BUA were downsampled to 512 Hz and the coherence and power were calculated using the Neurospec toolbox (Halliday et al., 1995) with a FFT size of 512, unless otherwise stated. This gives a frequency resolution of 1 Hz. The log power spectrum for each channel was z-scored relative to the mean and std. of the high frequency activity (100-150 Hz).

### 2.3 Signal processing of background unit activity (BUA) and beta envelope

Background unit activity (BUA) was derived from raw probe recordings in line with several previous studies (Moran and Bar-Gad, 2010; Sharott et al., 2017; Cagnan et al., 2019; Nakamura et al., 2021). First, we high-pass filtered at 300 Hz using a 3rd order Butterworth filter. Large spikes that could dominate the signal were identified by setting a threshold of mean ± 4 SD of the recording, and then a 4 ms segment around each instance crossing this threshold was removed and replaced with a randomly selected epoch which did not contain any spiking activity (Cagnan et al., 2019). Data was rectified and then low-pass filtered at 300 Hz using a third order Butterworth filter. BUA was downsampled to 1000 Hz for subsequent analysis. BUA was then filtered around ±5 Hz of the frequency of maximum coherence with the frontal ipsilateral ECoG in the beta frequency range (15-35 Hz), using a 2nd order Butterworth filter. For 85 % of channels the frequency of peak coherence with the frontal ipsilateral ECoG was within +/− 2 Hz of its peak power. The instantaneous envelope of beta oscillations was computed from the magnitude of the Hilbert transform of the beta filtered BUA. The change in the envelope of beta oscillations over 50 ms was calculated for each envelope-timeseries. The first and last 200 ms of data were removed to avoid any edge effects. On average, 2153.9 ± 893.5 (mean +/− std) data points (differenced envelope of beta filtered BUA) were computed per recording over the 45 recordings. All filtering was conducted using zero phase lag filters.

### 2.4 Beta ensemble identification

Beta ensembles were identified using a two-step statistical process, which allowed us to identify groups of channels with correlated changes in beta envelope (Lopes-dos-Santos et al., 2011; Lopes-dos-Santos et al., 2013; van de Ven et al., 2016).

#### 2.4.1 Principle component analysis

A matrix Z was constructed where the z-scored change in the envelope of beta filtered BUA over 50 ms for each channel made up a column. The matrix Z is, therefore, a T×N, where T is the number of 50 ms intervals that the change in envelope of the beta filtered BUA is calculated over and N is the number of channels.

Principal component analysis (PCA) was then applied to the matrix Z, with the intention of reducing the dimensionality from the number of channels to a smaller set of dimensions. To determine the number of significant patterns embedded in the data, each column of matrix Z had a random number of consecutive elements shifted from its end to its start, giving rise to the matrix $Z_{\mathrm{shift}}$. The data for each channel in the rows of $Z_{\mathrm{shift}}$, therefore, are no longer from the same point in time and any co-ordination detected arises due to chance. PCA was then performed on $Z_{\mathrm{shift}}$ and the resulting eigenvalues were stored. This process was repeated 1000 times to produce a distribution of eigenvalues under null conditions. A significance threshold was set using a Bonferroni correction for the number of principle components tested. Given that the number of principal components is equal to the number of channels included in analysis, $\;\alpha =\frac{0.05}{N}$. If an eigenvalue from PCA on Z was greater than the value of the distribution of eigenvalues produced from the shifted data at the $100\left(1-\alpha \right)\;\mathrm{th}$ percentile, the corresponding principal component was considered significant. Significant principal components were stored a N×S matrix, $P_{\mathrm{sign}}$, S is the number of significant principal components.

#### 2.4.2 Independent component analysis

To avoid constraining assembly patterns to be orthogonal and to allow the detection of higher-order correlations, independent component analysis (ICA) is used (Lopes-dos-Santos et al., 2011; van de Ven et al., 2016). Data is first projected onto the subspace spanned by the significant principal components, followed by ICA to prevent the detection of spurious patterns. The unmixing matrix was expressed in the original basis. This results in a N×S matrix, where N is the number of channels and S is the number of significant principal components. The columns of this matrix are the weight vectors of the assembly pattern. The sign and size of these weights are arbitrary, so each column of V was scaled to unit length, and the sign of each column of V was set such that its largest vector weight was positive. The background unit recorded by a channel was said to be a member of an ensemble if its absolute weight in the assembly pattern was greater than the 80th percentile of absolute weights. ICA was carried out using the fastICA algorithm by Gävert et al. (https://research.ics.aalto.fi/ica/fastica/).

#### 2.4.3 Raw-BUA ensembles

To determine the optimal input for the PCA-ICA pipeline, we repeated the above procedure but with raw-BUA instead of the change in the envelope of beta filtered BUA.

### 2.5 Eigenvalues and frequency

The relationship between the frequency band of BUA and the strength of the correlations identified by PCA were assessed by filtering the raw BUA at ±5 Hz using a second order Butterworth filter around a centre frequency varying from 8 Hz to 100 Hz in 1 Hz increments. The change in envelope was then computed and PCA was applied. The duration over which the change in envelope was computed was the period of the centre frequency, so the number of samples PCA was conducted on depended on the centre frequency of the oscillation. For instance, for filtering in the range 45-55 Hz, we would look at changes in envelope over 20 ms (1 / 50 Hz). However, the magnitude of eigenvalues that arise due to chance changes as a function of the number of samples included in PCA. To account for this, eigenvalues were normalized by subtracting and then dividing by the significance threshold for ensembles (see above) for that frequency band. The normalized eigenvalues therefore reflect proportionally how much greater an eigenvalue is than would be expected under null conditions.

The magnitude of eigenvalues and number of significant ensembles were compared when filtering the raw-BUA in the beta (15-25 Hz), theta (6-10 Hz), low-gamma (35-60 Hz) and high-gamma (60-90 Hz) frequency bands (filtering was carried out using a 2nd order Butterworth filter). Again, the change in envelope was calculated over the period of the centre frequency of each frequency band (e.g. a 125 ms period was used for theta oscillations (6-10 Hz), whereas a 13 ms period was used for high gamma oscillations (60-90 Hz)). Gamma oscillations have a shorter period than beta and theta oscillations which would result in more data points for both the low and high gamma band. To avoid variability in the magnitude of eigenvalues due to the number of data points, changes in the envelope of beta and gamma band oscillations were randomly subsampled without replacement to match the same number of data points as were available for theta oscillations. As a result, the same number of data points were subjected to PCA for each frequency band, making the eigenvalues comparable across conditions.

For both of the above analyses, we varied the timeframe used to measure the changes in envelope in line with the cycle duration corresponding to the centre frequency of the band-pass filter. Our reason for scaling the analysis window in this way was due to the difficulty in interpreting changes in the envelope over periods less than the wavelength of the filter frequency. Amplitude is often conceptualised as the peak to trough displacement of an oscillation. As such, it makes sense to think about amplitude as a property of at least one cycle of the oscillation. It is, therefore, most appropriate to measure changes in the amplitude at a timescale of one or more oscillation cycles.

### 2.6 Assembly expression strength

The expression strength of the assembly patterns was then calculated for each point in time (Lopes-dos-Santos et al., 2013, van de Ven et al., 2016):

$$R_{s}=z{\left(t\right)}^{T}P_{s}z\left(t\right)$$

where $z\left(t\right)$ is a vector whose elements are the z-scored change in envelope of beta filtered BUA over a sliding 50 ms window at time t for each of the channels and $P_{s}$ is the outer product of each column of V (s=1,…,S):

$$P_{s}={V_{s}}^{T}V_{s}$$

after the elements in the leading diagonal are set to 0.

Setting the leading diagonal to 0 ensures that a change in the envelope in the beta filtered BUA in a single channel with a large contribution to the assembly pattern is not sufficient to bring about high ensemble expression-strength. A threshold of 5 was set to define an ensemble activation, as has previously been used for ensembles of single units (van de Ven et al., 2016; Trouche et al., 2019). These activations were used as a trigger point for averaging the envelope and phase synchrony of beta oscillations.

### 2.7 Phase synchrony

The instantaneous phase of beta filtered (again using a centre frequency determined by the frequency of maximum coherence with the frontal ECoG +/− 5 Hz) BUA was derived from the Hilbert transform. The centre frequency of beta filtered BUA was highly consistent across channels within a recording. The centre frequency of 93 % of channels was within +/− 2 Hz of the median centre frequency for that recording. Phase synchrony index (PSI) was then computed for overlapping 50 ms intervals for pairs of channels a and b:

$$\mathrm{PS}I=\left|\left(\sum_{k=1}^{\tau }\exp\left(-i\left(\emptyset_{k}^{a}-\emptyset_{k}^{b}\right)\right)\right)\right|/\tau$$

where $\emptyset_{k}^{a}$ and $\emptyset_{k}^{b}\;$is the instantaneous phase of the beta filtered BUA of channel a and b respectively, and τ is the number of samples that make up the 50 ms windows (Cagnan et al., 2019). While non-overlapping windows were used for PCA-ICA to avoid inflating the magnitude of eigenvalues, here an overlapping window allows for greater temporal resolution into the changes in phase synchrony.

### 2.8 Correlations between signals

**Fig. 1:**
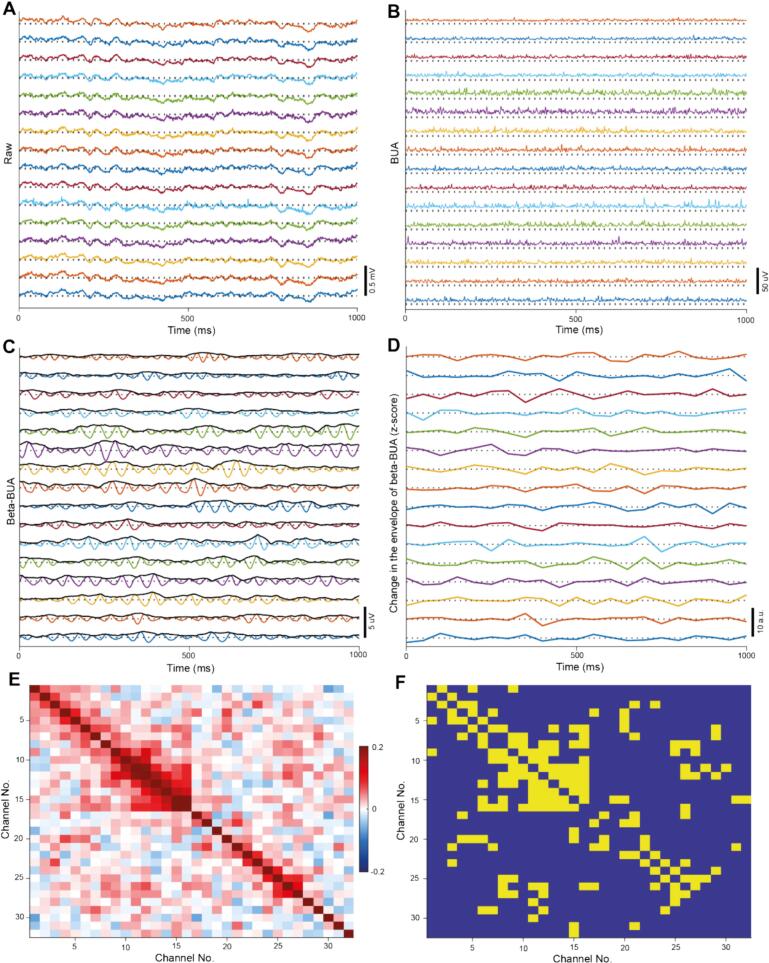
Analysing covariance in the change in envelope of beta-BUA within and across basal ganglia structures: Recordings were made with a pair of multi-electrode probes either both targeted to the GP or one targeted to the GP and the other to the STN. Either a single probe with two 16 channel shanks (targeted to the GP) or two probes each with 16 channels (targeted to the GP and STN) were used. Each shank in both configurations consisted of 16 electrodes arranged in a single vertical plane separated by 100 μm. A: The wideband signal recorded from 16 channels on a single recording shank in the GP. The dotted line represents zero for each signal. B: The BUA from 16 channels on a single recording shank in GP. The dotted line represents zero for each signal. C: The beta filtered BUA (coloured) and the envelope of this signal (black) from 16 channels on a single recording shank in the GP. The dotted line represents zero for each signal. D: The z-scored change in envelope of the beta filtered BUA over 50 ms from 16 channels on a single recording shank in the GP. The dotted line represents zero for each signal. E: An exemplary correlation matrix from a single recording session showing the correlations between the change in the envelope of beta filtered BUA over 50 ms windows for pairs of channels. The channels numbered 1–16 were from probe 1, whereas the channels numbered 17–32 were from probe 2. Channels are numbered by their position on the probe (e.g., channels 2 and 3 neighbour each other on probe 1). F: The significantly correlated pairs of channels (yellow) in the correlation matrix E. The main diagonal, which displays the correlation of a channel with itself, was displayed as non-significant in matrix F as significant correlations here are not meaningful.

Correlations between the change in beta envelope over 50 ms bins and cross correlations in the raw BUA in members and non-members were calculated across all permutations of pairs of channels and then averaged for each ensemble or recording. Similarly, when calculating the correlation or cross-correlation at a given distance, all permutations of pairs of channels at that distance were computed and then averaged for each ensemble or recording. Whether pairs were more positively correlated than expected due to chance (e.g., Fig. 1F) was assessed by circularly shuffling the change in the envelope of the beta filtered BUA for each pair of channels 1000 times. If the true correlation exceeded the 95th percentile for shuffled data a pair of channels was classified as significantly correlated.

### 2.9 Analysis and plotting

Analysis and plotting were carried out using custom written code in MATLAB (Mathworks, Natick, MA, USA). Error bars represent standard error of the mean (SEM) unless otherwise stated.

## 3 Results

Our overarching aim was to identify the spatial extent of basal ganglia unit activity that was coordinated by pathophysiological beta oscillations. Unlike LFPs, background unit activity (BUA), which reflects the synchronous spiking of the neural population proximal to the recording electrode, is a well spatially localised signal. Background unit activity (BUA) was computed from the GP and STN (Fig. 1A, B) of anaesthetised, parkinsonian rats (Mallet et al., 2008a; Cagnan et al., 2014). BUA in lesioned animals had a strong, stable oscillation in beta power (Fig. S1). A Hilbert transform was used to extract the envelope of the beta filtered BUA (Fig. 1C), the change in the envelope over 50 ms windows was then computed (Fig. 1D). This allowed us to detect changes in the amplitude of beta oscillations in the firing of neurons in the population of neurons proximal to the recording electrode. We focused on the correlations between the change in envelope across channels rather than the envelope itself, as it allowed us to identify groups of channels where the emergence and offset of beta oscillation occurred synchronously. Supporting the use of the change in beta envelope as the input to the PCA-ICA pipeline, PCA-ICA trained on the raw BUA did not result in ensembles which capture beta dynamics (Fig. S2).

We examined the change in beta-envelope over 50 ms as this is the approximate period of the beta oscillations observed here (centre frequency: 20.9 Hz ± 2.7 Hz). Moderate pairwise correlations were observed between the change in the envelope of beta filtered BUA (in channels across the GP and STN (Fig. 1E)). This was particularly the case for channels in close proximity, as indicated by the high correlations observed around the main diagonal of the correlation matrix. There were, also, several significant correlations observed over greater distances (Fig. 1F), including between channels on different recording probes. BUA is a far better localised signal, as compared with monopolar LFP, (Fig. S3) so these correlations are unlikely to result from volume conduction. This demonstrates beta oscillations emerge in a coordinated fashion across channels, including channels that are not immediately adjacent or on the same recording probe.

### 3.1 Synchrony and beta ensembles

**Fig. 2:**
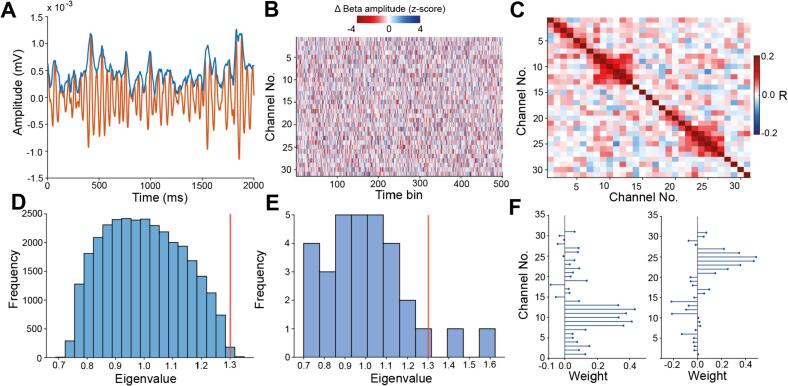
PCA-ICA was used to identify groups of channels with coordinated changes in the envelope of beta filtered BUA: All data presented here is from a single recording session in the GP. A: Exemplary beta-BUA (orange) and beta-envelope (blue) from a single probe channel in the GP. B: Exemplary z-scored change in beta-envelope over consecutive 50 ms windows for a single recording (only a section of the recording is shown here for display purposes). C: The correlation matrix of the z-scored change in beta envelope recorded over 31 channels in a single recording. All channels here were located in the GP. D: The distribution of eigenvalues from PCA applied to the matrix of z-scored changes in beta-envelope under null conditions (with randomly circularly shifted columns). The red line shows the significance threshold determined by a percentile cut-off of the circularly shuffled data with Bonferroni correction. E: PCA was applied to the z-scored change in beta-envelope. Two principal components were classified as significant here on the basis that their eigenvalues were greater than the 95th percentile (adjusted by the Bonferroni correction) of eigenvalues from the circularly shifted data (vertical red line). ICA was then applied to the z-scored change in the envelope of the beta filtered BUA projected onto the significant components to identify the assembly patterns of the 2 beta ensembles. F: The assembly patterns of 2 significant beta ensembles identified in this single recording session. Each recording channel has a weight in these assembly patterns which represents how large a contribution BUA recorded on that channel makes to the identified beta ensemble. (For interpretation of the references to colour in this figure legend, the reader is referred to the web version of this article.)

We hypothesized that unit activity across the basal ganglia would be coordinated by *multiple* beta ensembles. To test this, we sought to identify *groups* of BUA signals recorded from different probe contacts (beta ensembles) that had co-ordinated changes in their oscillation strength. This was achieved using a two-step statistical process, PCA followed by independent component analysis (ICA), whereby *groups* of channels with correlated changes in beta envelope (Fig. 2) could be identified. 85 such significant beta ensembles were identified over 45 recordings in 15 animals, each described by a weight vector containing the contribution of each channel (Fig. 2F) to the beta ensemble. Supporting the likely small contribution of volume conduction, beta ensembles could be detected even when channels were excluded to create a minimum spacing of 200 and 300 μm (Fig. S4A, B). The number of ensembles per channel was no different even as the spacing between channels increased (Fig. S4C).

**Fig. 3:**
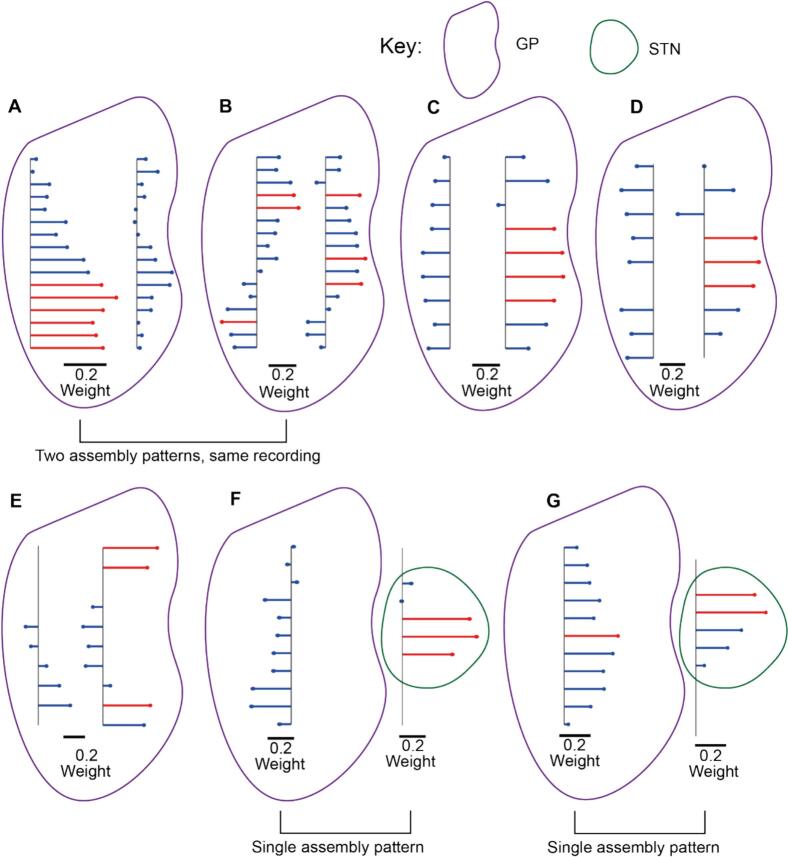
Beta ensembles were mostly spatially contiguous but could be non-contiguous, span shanks or even brain structures. A-E: Assembly patterns with a two-shank probe targeted to the GP. Member channels were coloured in red for display purposes. F, G: Assembly patterns with one probe targeted to the GP and another to the STN. Member channels were coloured in red for display purposes. (For interpretation of the references to colour in this figure legend, the reader is referred to the web version of this article.)

Channels with an absolute weight greater than the 80th percentile of the weight vector were classified as members of the beta ensemble (Fig. 3). In total, 85 beta ensembles were identified as significant on the basis of the magnitude of eigenvalues from PCA. In two recordings, the fastICA algorithm did not converge so these were excluded from analysis leaving 81 beta ensembles. Most recordings had probes only targeted to the GP (38/45) and as such 75 of the 81 ensembles were made up of channels in the GP alone. 6 of the 10 ensembles identified on the recording days with an STN recording probe contained at least 1 STN member, with 2 beta ensembles containing member channels both in the GP and STN. Member channels were generally clustered in space, although in some instances, member channels were separated by large distances (e.g., Fig. 3B, E and G). The majority of ensembles (57/81) were made up of member channels that were spatially contiguous. However, some examples (13/81) of ensembles with non-spatially contiguous members within a single probe could be identified, as could examples of ensembles which spanned the two shanks of the recording probe (12/81).

**Fig. 4:**
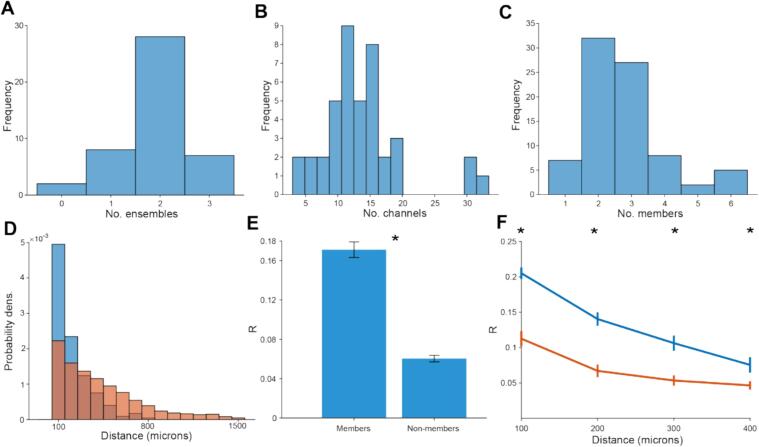
Beta ensembles were consistently identifiable across recordings and were made up of spatially clustered channels. A: A frequency histogram of the number of ensembles detected for each recording. B: A frequency histogram of the number of recording channels present in target structures (GP and STN) per recording. C: The frequency histogram of the number of member channels for each ensemble. D: The probability density histogram of the distance between pairs of member (blue) and non-member (red) channels on each recording probe individually. E: The average Pearson's R for changes in the envelope of beta filtered BUA was significantly greater for pairs of member channels than for pairs of non-member channels (Wilcoxon signed-rank test, *p* = 7.7 × 10^−14^). F: The average Pearson's R was greater between pairs of member channels (blue) than pairs of non-member channels (orange), separated by 100, 200, 300 or 400 μm (Wilcoxon rank sum test, *p* < 0.05 for 100, 200, 300 and 400 μm). (For interpretation of the references to colour in this figure legend, the reader is referred to the web version of this article.)

Strikingly, two beta ensembles were identified in most recordings (Fig. 4A), confirming that beta oscillations are not a completely unified process, but occur in spatially segregated ensembles. The number of channels within the GP or STN varied considerably across recordings (Fig. 4B), as did the number of member channels (Fig. 4C). For some analyses, the ensembles were discretised by taking those channels with the greatest contributions to an ensemble. This most frequently resulted in 2 or 3 member channels (Fig. 4C) although some were made up of larger numbers (up to 6). There were some instances were only a single member channel could be identified for an ensemble. This was a result of a short falling of using a percentile-based cut-off to define assembly membership. In recordings with small numbers of channels (i.e., less than 10), an 80th percentile cut-off did not consistently identify 2 or more sufficiently high weighted channels. None the less, these ensembles still represent a pattern of statistically significant coordination in the change in the envelope of beta filtered BUA, which undoubtedly involves the correlated activity of at least 2 channels. Member channels tended to be clustered spatially (Fig. 4D), with most member channels being separated by 500 μm or less.

Post-hoc correlation of the change in envelope of beta filtered BUA was greater between pairs of member channels than pairs of non-member channels (Fig. 4E), verifying that our method identified channels with coordinated beta onset and offset. This was still the case when comparing correlations between channels separated by the same distance (Fig. 4F), so this effect is not solely driven by member channels being more proximal than non-members. Some highly weighted channels had a negative weighting in the assembly pattern, indicating that power changes in that channel were in the opposite direction to other channels. However, these instances were rare (9/228 channels that were assembly members) so were excluded from the analysis. Whilst not mathematically prohibited by the PCA-ICA pipeline, only a small minority of channels were members of 2 or more ensembles (5/219 positively weighted members). Due to the relatively small number of STN recordings, we limited subsequent analysis to sessions where recordings were only made in the GP (71 beta ensembles) to simplify interpretation.

**Fig. 5:**
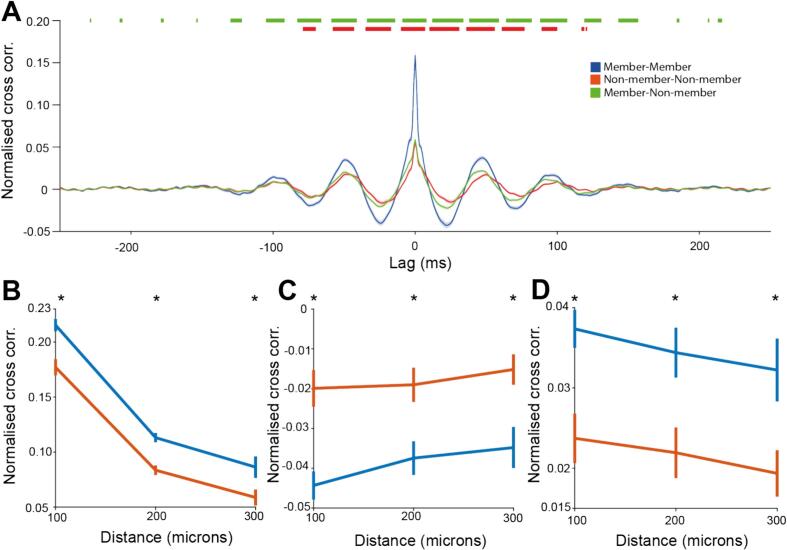
Members of beta ensembles showed correlated changes in the envelope of beta filtered BUA and increased beta band coherence. Cross correlation was normalized here such that the autocorrelation at lag 0 was 1. A: The normalized cross correlation between pairs of member channels (blue), pairs of non-member channels (orange) and pairs of member and non-member channels (green), at lags of -250 ms to 250 ms. Orange markers above the graph show significance between the cross correlation of pairs of members and pairs of non-member channels. Green markers above the graph show significance between the cross correlation of pairs of members and pairs of member and non-member channels. Significance was determined with the Wilcoxon rank sum test (member-member pairs vs non-member-non-member pairs) and the Wilcoxon signed-rank test (member-member pairs vs member-non-member pairs) using false discovery rate statistics to control for the multiple time points compared. B: The normalized cross correlation at a 0 ms lag between pairs of member channels (blue) was greater than that for pairs of non-member channels (orange) when separated by the distance between channels (Wilcoxon rank sum test, *p* < 0.003 for 100, 200 and 300 μm). C:The normalized cross correlation at a 25 ms lag between pairs of member channels (blue) was more negative than that of pairs of non-member channels (orange) when separated by the distance between channels (Wilcoxon rank sum test, *p* < 0.004 for 100, 200 and 300 μm). D: The normalized cross correlation at a 50 ms lag between pairs of member channels (blue) was greater than that for pairs of non-member channels (orange) when separated by the distance between channels (Wilcoxon rank sum test, *p* < 0.006 for 100, 200 and 300 μm). (For interpretation of the references to colour in this figure legend, the reader is referred to the web version of this article.)

The member channels identified in all analysis up to this point were identified by changes in the *amplitude* of beta envelope. We hypothesized that *phase-synchrony* between members of beta ensembles would also be higher than non-members, which would indicate that they are also coordinated on the timescale of individual cycles. To address this, we explored cross-correlations between *unfiltered* BUAs calculated between ensemble members and non-members. If correlations in the beta power between channels predicted beta synchrony, beta fluctuations should emerge through summation, without the need to filter (see Cagnan et al., 2019). Cross-correlations between raw BUA showed a peak at 0 ms lag and broader peaks at ±50 ms (Fig. 5A). The cross-correlation between members was greater than that between non-members at lags of 0 ms and lags of multiples of 50 ms corresponding to beta-frequency correlation (Fig. 5A), confirming that there is greater beta phase-synchronisation between member channels.

The cross correlation at 0 ms in raw BUA between pairs of member and pairs of non-member channels fell with increasing distance (Fig. 5B) but was greater between member channels than between non-members even after taking distance into account (Fig. 5B). Equally, the trough at 25 ms was lower (Fig. 5C) and the peak at 50 ms was higher (Fig. 5D) in member channels as compared to non-members after controlling for the distance between channels. This indicates that differences in phase-synchrony between members and non-members is not simply a function of the average distance between channels.

### 3.2 Beta ensembles were preferentially observed in the beta-band

**Fig. 6:**
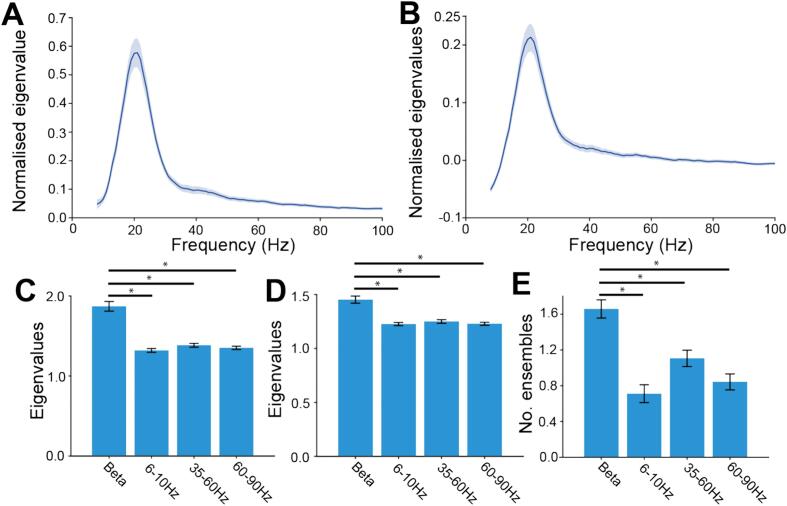
Coordinated oscillation in BUA was specific to the beta frequency band: A and B: The largest (A) and the average of the 3 largest (B) normalized eigenvalues from the PCA of the change in z-scored envelope of BUA filtered at ±5 Hz of the centre frequency. C, D and E: The change in envelope of beta oscillations (15-25 Hz) was compared to theta (6-10 Hz), low (35–60 Hz) and high (60–90 Hz) gamma in terms of the size of the first eigenvalue (C, Wilcoxon signed-rank test, beta-theta: *p* < 10^−6^, beta-low gamma: p < 10^−6^, beta-high gamma: p < 10^−7^), the average of the first 3 eigenvalues (D, Wilcoxon signed-rank test, beta-theta: p < 10^−6^, beta-low gamma: p < 10^−6^, beta-high gamma: p < 10^−7^), and the number of ensembles identified (E, Sign test, beta-theta: p < 10^−7^, beta-low gamma: p < 10^−4^, beta-high gamma: p < 10^−6^).

Given that filtering is a key part of the signal processing pipeline, it is possible that the results described thus far were due to covariance in wideband, rather than beta band amplitude. To address this, we compared the core analytical variables to those computed on other frequency bands. This was carried out by filtering the *raw* BUA in around +/− 5 Hz of increasing centre frequencies. The period that the change in envelope was calculated over was determined by the centre frequency of the oscillation. For instance, for a centre frequency of 40 Hz, the period the change in envelope was considered over would be 25 ms (1 / 40 Hz). The normalized variance explained by the first principal component was greater in the beta frequency range than in lower or higher frequency ranges (Fig. 6A), with a peak at ∼20 Hz. This was also true of the mean of the normalized variance explained by the first 3 principal components (Fig. 6B).

Next, we considered the coactivity across a number of frequency bands. To achieve this, we filtered raw-BUA in the theta (6-10 Hz), beta (15-25 Hz), low gamma (35-60 Hz) and high gamma (60-90 Hz) band. Again, the period that the change in envelope was considered over was determined by the centre frequency of the oscillation (e.g., for the theta band we considered the change in envelope over 125 ms, whereas for high gamma the change in envelope was calculated over 13 ms). The eigenvalue of the first principal component (Fig. 6C) and mean of the eigenvalues for the first three principal components (Fig. 6D) was significantly greater for beta frequency than theta or low or high gamma filtered BUA. In addition, significantly more beta ensembles could be identified in the beta band than for theta or low or high gamma bands (Fig. 6E). Therefore, oscillations at beta frequency led to more spatially coordinated neuronal activity than oscillations at other frequency bands in parkinsonian animals. Equally, no beta-ensembles could be detected in unlesioned controls, where there were no strong beta oscillations (Fig. S5).

### 3.3 Spatially coordinated beta emergence across time

**Fig. 7:**
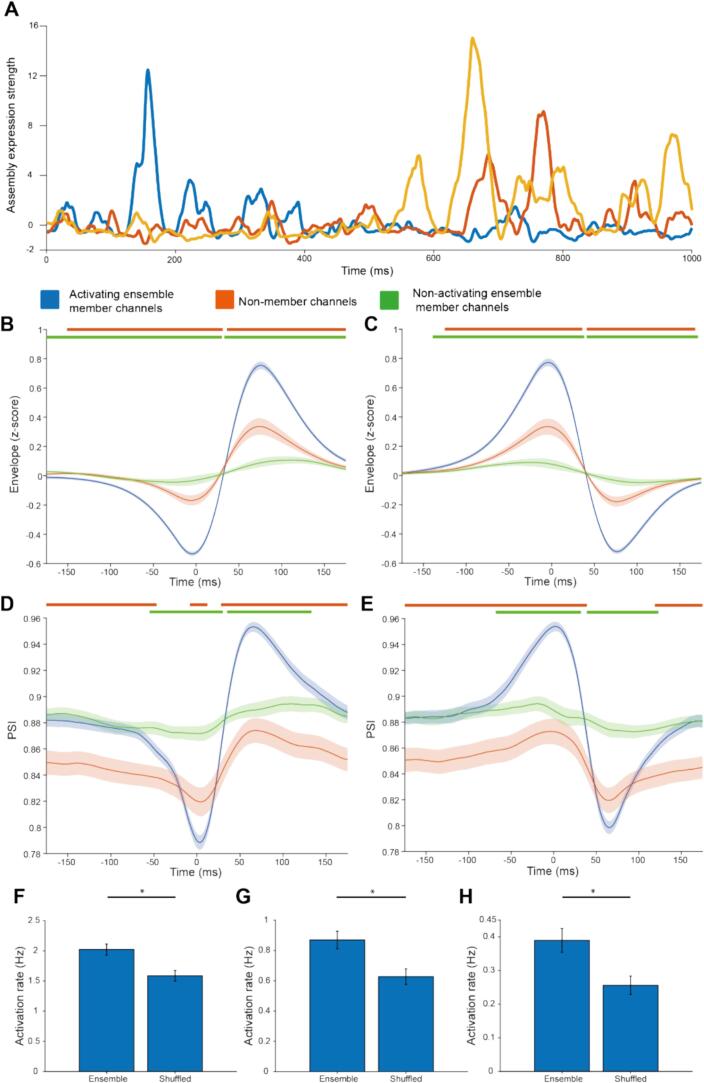
Coactive changes in beta envelope in beta ensembles were associated with increases in phase synchrony specifically in member channels: A: The expression strength of the coactivity patterns of 3 ensembles (identified in a single recording) over time. B and C: The average beta-envelope triggered by ensemble activations (activation threshold = 5), separating activations by whether they were associated with a coordinated increase (B) or decrease (C) in beta envelope. These were associated with a larger coordinated increase and decrease in beta-envelope for B and C respectively in member channels of the activating (blue) than non-activating (green) ensembles or non-member channels (orange). D and E: The average PSI triggered by ensemble activations (activation threshold = 5), separating activations by whether they were associated with a coordinated increase (D) or decrease (E) in beta envelope. These were associated with a larger coordinated increase and decrease in PSI for D and E respectively in member channels of the activating (blue) than non-activating (green) ensembles or non-member channels (orange). For figs. B, C, D and E periods of significant difference are marked above the graph. The orange marker signifies periods of significant difference between members of the activating ensemble (blue) and non-member channels (orange). The green marker signifies periods of significant differences between members of activating (blue) and non-activating (green) ensembles. Significance was determined with the Wilcoxon rank sum test using false discovery rate statistics to control for the multiple time points compared. F, G and H: The frequency of activations was greater in ensembles compared with circularly shifted ensembles, with an activation threshold of 5 (Wilcoxon signed-rank test, *p* = 1.7 × 10^−10^), 7.5 (Wilcoxon signed-rank test, *p* = 8.8 × 10^−11^) and 10 (Wilcoxon signed-rank test, *p* = 2.5 × 10^−10^) respectively. (For interpretation of the references to colour in this figure legend, the reader is referred to the web version of this article.)

We next sought to understand the changes in instantaneous beta amplitude and phase sychrony across space during spatially coordinated beta emergence and offset. The expression strength of coactivity patterns was computed (Fig. 7A). Peaks in expression strength (beta ensemble activations) were primarily driven by large co-ordinated changes in the envelope of beta-BUA in at least a pair of channels with high contributions to the beta ensemble (Fig. 7B-C). The low stable baseline punctuated by large peaks reflected that much of the coordinated change in beta envelopes occurred as brief, transient events. Whilst these activation events were solely identified on the basis of changes in envelope, they were also accompanied by large changes in phase synchrony between member channels (Fig. 7D-E). These activations were significantly more frequent in true beta ensembles than in pseudo-beta ensembles formed by circularly shifting the assembly pattern for each beta ensemble by a random amount (Fig. 7F-H). This difference in activation rate between beta ensembles and pseudo-beta ensembles became increasingly pronounced as the threshold for activation was increased. Taken together, these findings confirm that coactivity in the emergence of beta oscillations is specific to the identified beta ensembles and is accompanied by an increase in phase synchrony.

### 3.4 Subcortical ensembles and their relationship with respect to ECoG

**Fig. 8:**
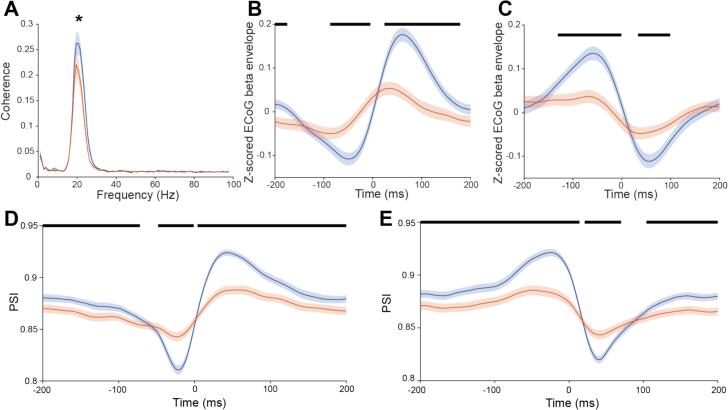
Beta ensemble activations were associated with changes in both the instantaneous power of cortical beta and the phase synchrony in the beta-band between the beta ensemble and the cortex: A: The peak coherence of the BUA of member channels (blue) with ECoG was greater than that of non-member channels (orange) (Wilcoxon rank sum test, *p* < 0.05). The coherence was calculated between ECoG and BUA sampled at 1000 Hz with a segment length of 1024. B and C: Average beta-ECoG envelope triggered by ensemble activations (blue) separating by whether these activations reflected a coordinated increase (B) or decrease (C) in the envelope of beta-BUA respectively. Also plotted was the triggered average of beta-ECoG envelope at random time points (orange) separating for whether these random time points correspond to a coordinated increase (B) or decrease (C) in the envelope of beta-BUA in the beta ensemble. D and E: PSI between beta-ECoG and beta-BUA averaged across ensemble activations in member (blue) and non-member (orange) channels, separating by whether these activations reflected a coordinated increase (D) or decrease (E) in the envelope of beta-BUA respectively. B, C, D and E have periods of significant difference between traces displayed with a black marker. Significance was determined with the Wilcoxon rank sum test using false discovery rate statistics to control for the multiple time points compared. (For interpretation of the references to colour in this figure legend, the reader is referred to the web version of this article.)

Cortical beta-bursts have been associated with increases in beta envelope in the GP and STN and increased phase synchrony between basal ganglia structures and the cortex (Cagnan et al., 2019). Previous work has probed the relationship between ECoG and the basal ganglia at a brain structure-by-structure level. We aimed to test whether beta neural populations in the GP that showed coordinated beta oscillation emergence had a different relationship with cortical beta oscillations to non-members. Both member and non-member channels had a large rise in their coherence with ECoG at ∼15–30 Hz, which peaked at ∼20.5 Hz. This peak in coherence was larger with BUA channels that were ensemble members (Fig. 8A). Ensemble activations were divided by whether they represented a coordinated increase or decrease in the envelope of beta-BUA. Ensemble activations were associated with moderate changes in the envelope of beta-ECoG (Fig. 8B, C). This moderate amplitude likely reflects that some, but not all, ensemble activations are associated with beta bursts in the cortex. Equally, the change in PSI across ensemble activations between beta-ECoG and beta-BUA in member channels was greater than for channels that were not members of the activating beta ensemble (Fig. 8D, E). Overall, membership of a beta ensemble predicted that a given channel was more strongly related to cortical beta.

## 4 Discussion

**Fig. 9:**
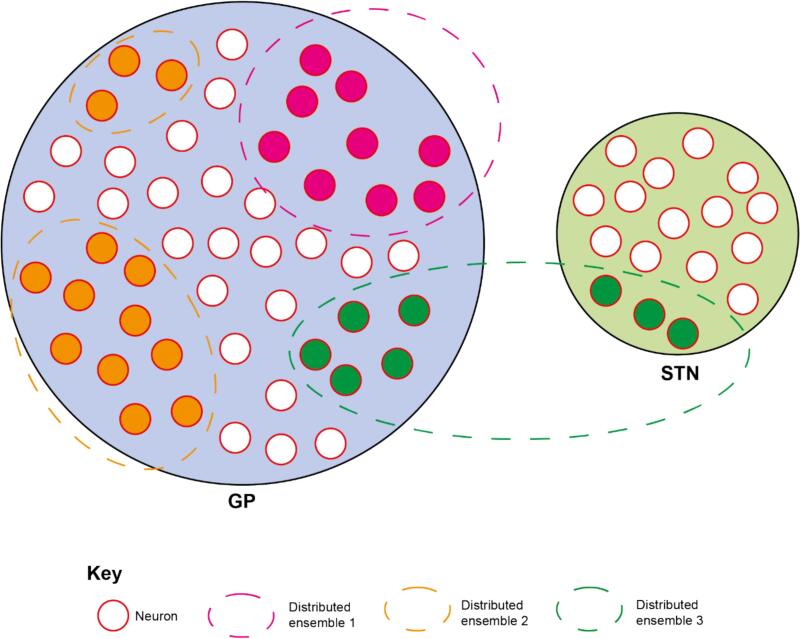
Neurons across the GP and STN form distinct beta ensembles which each have coordinated changes in the envelope of their beta oscillations. Beta oscillations evolve synchronously across the neural population that makes up a beta ensemble, which can form within or across anatomical structures. As coordinated beta activity is accompanied by an increase in beta phase synchrony in the neurons that make up beta ensembles, they likely indicate dynamic synchronisation of spike timing across large spatial extents of the basal ganglia.

Abnormally sustained, transient increases in the power of beta oscillations are markers of impaired network activity in the parkinsonian brain. How these transient increases in beta synchrony manifest on the spatial scale, however, is less clear. Here we provide a novel application of a well-established analytical technique to define the nature of spatial synchronisation in multichannel unit recordings from parkinsonian rats. The results demonstrate that the spiking of multiple, independent ensembles of neurons can be distinctly and transiently synchronized by beta oscillations within the cortico-basal ganglia network (Fig. 9). These findings have potentially important implications for the interpretation of beta oscillations as a biomarker of pathophysiology in PD and its treatment with DBS.

### 4.1 Anatomical properties of cortico-basal ganglia circuits and beta oscillations

An influential hypothesis as to the pathophysiological mechanism in PD is that the functional segregation of cortico-basal ganglia loops is compromised (Filion et al., 1988; Tremblay et al., 1989; Boraud et al., 2000; Leblois et al., 2006), preventing the normal functioning of those circuits. Cortico-basal ganglia circuits are arranged into information streams that can be broadly divided by cortical inputs to the striatum (Alexander et al., 1990). These were traditionally thought to consist of 3/4 functional subdivisions, but recent studies have shown that in rodents there are 10s–100s of cortico-basal ganglia-thalamic loops in the mouse brain (Hunnicutt et al., 2016; Peters et al., 2021). Inputs from functionally related cortical areas converge into specific parts of the striatum, but these clusters remain segregated in their axonal projections to the pallidum (Foster et al., 2021). However, there is significant convergence of these striatopallidal channels onto the basal ganglia output nuclei and the subsequent thalamocortical projection. Overall, cortico-basal ganglia circuits are, therefore, partially segregated with multiple points of convergence. The expression and propagation of beta oscillations will be partly defined by these anatomical features. As previously suggested, the spatial hypersynchronization of beta oscillations could bind the activity of neurons across usually segregated loops, constraining the computation space (Brittain et al., 2014; Cagnan et al., 2015). To our knowledge, however, the spatial extent of such hypersynchronization has not been systematically quantified. By providing an approach to quantify the dependence of beta oscillations across space, we aimed to provide novel insights into this issue.

### 4.2 Defining spatial synchronisation of beta oscillations at the neuronal level

We analysed the temporal correlation of the change in beta amplitude of BUA signals recorded from equally spaced recording channels on silicon probes. We chose the power of the BUA as our primary signal for analysis because it corresponds to the level of beta synchronisation of spiking in a small pool of neurons around the electrode (Sharott et al., 2017; Cagnan et al., 2019). This is a useful signal given that transient increases in beta synchronisation appear to be a key pathophysiological feature of the disease (Tinkhauser et al., 2017a; Cagnan et al., 2019; Baaske et al., 2020). When the fluctuations in BUA beta amplitude are positively correlated across electrode channels, it suggests that the same process is coordinating local synchronisation across those areas. For analysis of such temporal correlation to be meaningful, it is important that the synchronisation of signals results from neuronal processes, rather than passive ones such as volume conduction. LFPs are therefore suboptimal for this purpose, as re-referencing cannot guarantee independence and more complex signal processing complicates interpretation. In contrast, the change in envelope of beta-BUA signals have a high level of independence without further referencing, with even adjacent channels outside of the beta ensembles explaining an average of only approximately 1 % of each other's variance. Thus, when fluctuations in the envelope of beta-BUA are significantly correlated, it is likely to be driven by neuronal activity.

To quantify these correlations across every channel in a given recording, we employed a PCA/ICA framework that is well-established for the study of single units (Peyrache et al., 2010; Lopes-dos-Santos et al., 2011; van de Ven et al., 2016; Lopes-dos-Santos et al., 2013). The key feature of this method is that it defines *groups* of channels with correlated changes in the amplitude of beta oscillations in their spiking activity that are otherwise independent of each other and is agnostic to distance. Interestingly, the expression strength of assembly patterns tended to have a stable baseline with transient peaks. This mirrors findings from ensembles identified in single units, where again coordinated firing is dominated by short duration events (Lopes-dos-Santos et al., 2013, van de Ven et al., 2016). These peaks likely represent coordinated beta bursts across beta ensembles. In the majority of recordings, this method detected more than one ensemble. This result suggests that beta synchronisation is not a homogenous process across circuits, but there are multiple, distinct beta “activities” that can couple across specific neuronal populations independently of each other. This highlights the complementarity of our approach too more traditional pairwise methods, which do not lend themselves to finding such independent groups of channels. In our experimental set-up, we cannot identify the substrate for such independent beta activities, but it seems likely that they could be the result of the anatomical segregation of basal ganglia circuits described above. Importantly, many ensembles were not spatially contiguous and/or spanned the pallidum and STN. Ensembles may therefore have represented pools of neurons connected by common inputs, rather than simply being in direct proximity to each other.

### 4.3 Physiological properties of beta ensembles

The ensembles defined by our analysis were based on fluctuations in beta amplitude, but were accompanied by enhanced beta phase synchronisation with other ensemble members and the cortex. This beta synchronisation is likely to be the cause, rather than effect of coordinated fluctuations in beta power across channels. We have already described the transient increase in phase locking of individual units/BUA channels during cortical beta bursts (Cagnan et al., 2019). Here, we extend those observations to show that cortical beta bursts are accompanied by transient beta synchronisation across spatially distinct populations of basal ganglia neurons. In addition, the raw BUAs (i.e. not beta filtered) of member channels were around 100 % more correlated with each other at zero lag than with non-members. This indicates that groups of neurons that were spatially synchronized on the scale of beta oscillations were also far more likely to fire synchronous spikes within a few milliseconds of each other (Buzsaki, 2010). This finding is important, as it indicates that ensembles will produce highly synchronized outputs that are likely to propagate to, and temporally summate within, downstream targets. More speculatively, correlations on these time scales could contribute to maladaptive plasticity in the parkinsonian brain (Chu et al., 2015). On a technical level, using phase-synchronisation or cross correlation between channels to define ensembles may have led to similar results as using beta amplitude. However, changes in beta power provides a more straightforward signal for ICA/PCA and can readily be applied in other contexts for future studies. More importantly, our method provided an effective way of identifying spatially distributed channels that shared features relating to oscillation and synchronisation.

One potential caveat of our findings is the inability to fully distinguish two possible mechanisms through which the coordination reported here could arise. Mechanism 1: Beta ensembles reflect the coordinated emergence of beta oscillation in spiking activity of spatial extents of the basal ganglia made up of neurons proximal to each of the recording contacts. This is the interpretation we think is most likely. Mechanism 2: That beta ensembles are driven by large oscillations in the spiking output of focal neural ensembles that are detectable across multiple channels. In mechanism 2, PCA-ICA could be thought of as identifying ‘sources’ of beta oscillations. In this interpretation, several such distinct ‘sources’ can be identified within and across anatomical structures. Therefore, regardless which of the two mechanisms outlined above are at play, this paper provides evidence that there are neural populations within the basal ganglia with distinct emergence in relation to beta activity, be this spatial extents of the basal ganglia or more focal neural populations with oscillations large enough to be recorded across multiple contacts. In our opinion, Mechanism 1 remains the more likely of the two interpretations, as members of beta assemblies are more correlated even after controlling for distance. These correlations were seen up to 400 μm, a distance where it is highly unlikely the same spiking activity can be recorded. Assembly patterns identified could also be spatially non-contiguous, span recording probes and even anatomical structures.

### 4.4 Relevance to PD pathophysiology and DBS

As the recordings used here were made in anaesthetized rats, we cannot guarantee the generalisability of our findings to the awake state. There is a general tendency of anaesthesia to synchronise neural activity across the forebrain (Steriade, 2000), this could increase the synchronisation of beta ensembles as compared to awake animals. This is mitigated in part by the fact recordings were made in “cortical activation,” a state that resembles the awake brain more that the sleep-like slow-wave state (Mallet et al., 2008b; Mallet et al., 2008c). Beta oscillations under this anaesthetic protocol also generally have a lower centre frequency, which is more stable (see Fig. S1) than those recorded in awake animals (Delaville et al., 2015; Brazhnik et al., 2016; McNamara et al., 2022). However, given the general agreement between the properties of parkinsonian oscillations across the cortico-basal ganglia network in anaesthetised and awake rats (e.g. Sharott et al., 2005; Mallet et al., 2008c for STN), it seems reasonable to assume that the core observations here may also translate to awake animals. Taking the caveats into account, the principles identified here raise specific hypotheses as to how spatial synchronisation of beta activities are related to Parkinsonian symptoms. Notably, it seems reasonable to speculate that the expression of ensembles would change during movement and/or dopamine replacement. Given that beta synchronisation is generally reduced during these conditions (Hammond et al., 2007), we hypothesise that ensemble expression would decrease and/or ensembles would decrease in size/become more segregated. High-density recordings in awake animals would provide and ideal experimental set-up to test these hypotheses.

Beta oscillations, quantified in a variety of ways, are most strongly related to akinetic/rigid symptoms (Kühn et al., 2005; Sharott et al., 2014; Neumann et al., 2016). Within each patient, the severity of akinetic rigid symptoms often varies between upper and lower body and individual limbs. Our findings suggest that independently synchronized ensembles could underlie this variance and provide a mechanism through which different limbs could become bradykinetic at a given time. This naturally leads to the hypothesis that dopaminergic medication and the consequent reduction in akinetic/rigid symptoms would lead to smaller and/or more spatially segregated ensembles. Performing similar experiments in awake animals with silicon probes would allow this hypothesis to be fully tested.

Adaptive DBS targeting beta bursts has been found to outperform continuous stimulation in patients with Parkinson's disease (Little et al., 2013). This allows large reductions in the electrical energy that must be delivered to control symptoms, while reducing the rate of battery depletion and the severity of side effects (Little et al., 2013). Stimulation is typically delivered with a single stimulating electrode within a single brain structure (i.e., STN or GP, not both). Given that spatially clustered subregions in the basal ganglia show coordinated changes in the envelope of beta activity, there may also be value to spatial targeting for adaptive DBS protocols. Such a stimulation strategy was pioneered by Tass and colleagues, where electrical stimulation has been delivered from different contacts of a stimulating electrode in an open loop fashion to reset the coordinated neural activity observed in Parkinsonism (Tass, 2003; Tass et al., 2012). The main advantages of coordinated reset over other adaptive stimulation approaches are long term plastic changes and delayed symptom return (Tass et al., 2012). As discussed above, ensembles in our data were synchronized at timescales relevant for the induction of plasticity. More targeted interference of such spatiotemporal synchronisation thus has the potential to interfere with this process and could result in larger long-term effects on symptom expression. The use of segmented leads could potentially facilitate such an approach (Debove et al., 2023). Future work may focus on combining both adaptive stimulation approaches and deliver targeted bursts of stimulation to spatiotemporally distinct pockets of ensemble activity to further reinforce the desynchronizing effects of stimulation and potentially induce longer lasting plastic changes.

## Funding statement

This work was supported by 10.13039/501100000265Medical Research Council UK Awards MR/R020418/1 and  MR/X023141/1 (to H.C.), U138197109, MC_UU_12020/5, MC_UU_12024/2 and MC_UU_00003/5 (to P.J.M.), and MC_UU_12024/1 and MC_UU_00003/6 (to A.S.); 10.13039/501100000304Parkinson's UK Grant G-0806 (to P.J.M.).

## CRediT authorship contribution statement

**Isaac Grennan:** Writing – review & editing, Writing – original draft, Methodology, Formal analysis, Conceptualization. **Nicolas Mallet:** Writing – review & editing, Investigation, Data curation. **Peter J. Magill:** Writing – review & editing, Investigation, Funding acquisition. **Hayriye Cagnan:** Writing – review & editing, Writing – original draft, Supervision, Funding acquisition, Formal analysis, Conceptualization. **Andrew Sharott:** Writing – review & editing, Writing – original draft, Supervision, Methodology, Funding acquisition, Conceptualization.

## Declaration of competing interest

The authors declare the following financial interests/personal relationships which may be considered as potential competing interests:

Andrew Sharott reports financial support and article publishing charges were provided by UK Research and Innovation Medical Research Council. Hayriye Cagnan reports financial support was provided by UK Research and Innovation Medical Research Council. Peter Magill reports financial support was provided by UK Research and Innovation Medical Research Council. Peter Magill reports financial support was provided by Parkinson's UK. If there are other authors, they declare that they have no known competing financial interests or personal relationships that could have appeared to influence the work reported in this paper.

## Data Availability

The preprocessed data is available at: https://data.mrc.ox.ac.uk/data-set/wideband-recordings-silicon-probes-subthalamic-nucleus-6-ohda-hemi-lesioned-rats-during and has DOI: https://doi.org/10.5287/bodleian:wx6D7oenk

## Appendix A Supplementary data

Supplementary material Image 1

## References

[bb0005] Alexander G.E., Crutcher M.D., DeLong M.R. (1990). Basal ganglia-thalamocortical circuits: parallel substrates for motor, oculomotor, “prefrontal” and “limbic” functions. Prog. Brain Res..

[bb0010] Baaske M.K., Kormann E., Holt A.B., Gulberti A., McNamara C.G., Potter-Nerger M., Westphal M., Engel A.K., Hamel W., Brown P., Moll C.K.E., Sharott A. (2020). Parkinson’s disease uncovers an underlying sensitivity of subthalamic nucleus neurons to beta-frequency cortical input in vivo. Neurobiol. Dis..

[bb0015] Boraud T., Bezard E., Bioulac B., Gross C.E. (2000). Ratio of inhibited-to-activated pallidal neurons decreases dramatically during passive limb movement in the MPTP-treated monkey. J. Neurophysiol..

[bb0020] Bracklein M., Barsakcioglu D.Y., Del Vecchio A., Ibanez J., Farina D. (2022). Reading and modulating cortical beta bursts from motor unit spiking activity. J. Neurosci..

[bb0025] Brazhnik E., McCoy A.J., Novikov N., Hatch C.E., Walters J.R. (2016). Ventral medial thalamic nucleus promotes synchronization of increased high Beta oscillatory activity in the basal ganglia-Thalamocortical network of the Hemiparkinsonian rat. J. Neurosci..

[bb0030] Brittain J.S., Sharott A., Brown P. (2014). The highs and lows of beta activity in cortico-basal ganglia loops. Eur. J. Neurosci..

[bb0035] Brown P. (2007). Abnormal oscillatory synchronisation in the motor system leads to impaired movement. Curr. Opin. Neurobiol..

[bb0040] Brown P., Oliviero A., Mazzone P., Insola A., Tonali P., Di Lazzaro V. (2001). Dopamine dependency of oscillations between subthalamic nucleus and pallidum in Parkinson’s disease. J. Neurosci..

[bb0045] Buzsaki G. (2010). Neural syntax: cell assemblies, synapsembles, and readers. Neuron.

[bb0050] Buzsaki G., Anastassiou C.A., Koch C. (2012). The origin of extracellular fields and currents--EEG, ECoG, LFP and spikes. Nat. Rev. Neurosci..

[bb0055] Cagnan H., Kuhn A.A., Brown P. (2014). Co-modulation of finely tuned high-gamma band activity across hemispheres in Parkinson’s disease. Clin. Neurophysiol..

[bb0060] Cagnan H., Duff E.P., Brown P. (2015). The relative phases of basal ganglia activities dynamically shape effective connectivity in Parkinson’s disease. Brain.

[bb0065] Cagnan H., Mallet N., Moll C.K.E., Gulberti A., Holt A.B., Westphal M., Gerloff C., Engel A.K., Hamel W., Magill P.J., Brown P., Sharott A. (2019). Temporal evolution of beta bursts in the parkinsonian cortical and basal ganglia network. Proc. Natl. Acad. Sci. USA.

[bb0070] Chu H.Y., Atherton J.F., Wokosin D., Surmeier D.J., Bevan M.D. (2015). Heterosynaptic regulation of external globus pallidus inputs to the subthalamic nucleus by the motor cortex. Neuron.

[bb0075] Debove I., Petermann K., Nowacki A., Nguyen T.K., Tinkhauser G., Michelis J.P., Muellner J., Amstutz D., Bargiotas P., Fichtner J., Schlaeppi J.A., Krack P., Schuepbach M., Pollo C., Lachenmayer M.L. (2023). Deep brain stimulation: when to test directional?. Mov. Disord. Clin. Pract..

[bb0080] Delaville C., McCoy A.J., Gerber C.M., Cruz A.V., Walters J.R. (2015). Subthalamic nucleus activity in the awake hemiparkinsonian rat: relationships with motor and cognitive networks. J. Neurosci..

[bb0085] Engel A.K., Fries P., Singer W. (2001). Dynamic predictions: oscillations and synchrony in top-down processing. Nat. Rev. Neurosci..

[bb0090] Feingold J., Gibson D.J., DePasquale B., Graybiel A.M. (2015). Bursts of beta oscillation differentiate postperformance activity in the striatum and motor cortex of monkeys performing movement tasks. Proc. Natl. Acad. Sci. USA.

[bb0095] Filion M., Tremblay L., Bedard P.J. (1988). Abnormal influences of passive limb movement on the activity of globus pallidus neurons in parkinsonian monkeys. Brain Res..

[bb0100] Foster N.N. (2021). The mouse cortico-basal ganglia-thalamic network. Nature.

[bb0105] Halliday D.M., Rosenberg J.R., Amjad A.M., Breeze P., Conway B.A., Farmer S.F. (1995). A framework for the analysis of mixed time series/point process data--theory and application to the study of physiological tremor, single motor unit discharges and electromyograms. Prog. Biophys. Mol. Biol..

[bb0110] Hammond C., Bergman H., Brown P. (2007). Pathological synchronization in Parkinson’s disease: networks, models and treatments. Trends Neurosci..

[bb0115] Hunnicutt B.J., Jongbloets B.C., Birdsong W.T., Gertz K.J., Zhong H., Mao T. (2016). A comprehensive excitatory input map of the striatum reveals novel functional organization. Elife.

[bb0120] Kühn A.A., Trottenberg T., Kivi A., Kupsch A., Schneider G.H., Brown P. (2005). The relationship between local field potential and neuronal discharge in the subthalamic nucleus of patients with Parkinson’s disease. Exp. Neurol..

[bb0125] Kuhn A.A., Kempf F., Brucke C., Gaynor Doyle L., Martinez-Torres I., Pogosyan A., Trottenberg T., Kupsch A., Schneider G.H., Hariz M.I., Vandenberghe W., Nuttin B., Brown P. (2008). High-frequency stimulation of the subthalamic nucleus suppresses oscillatory beta activity in patients with Parkinson’s disease in parallel with improvement in motor performance. J. Neurosci..

[bb0130] Leblois A., Meissner W., Bezard E., Bioulac B., Gross C.E., Boraud T. (2006). Temporal and spatial alterations in GPi neuronal encoding might contribute to slow down movement in parkinsonian monkeys. Eur. J. Neurosci..

[bb0135] Little S., Pogosyan A., Neal S., Zavala B., Zrinzo L., Hariz M., Foltynie T., Limousin P., Ashkan K., FitzGerald J., Green A.L., Aziz T.Z., Brown P. (2013). Adaptive deep brain stimulation in advanced Parkinson disease. Ann. Neurol..

[bb0140] Lopes-dos-Santos V., Conde-Ocazionez S., Nicolelis M.A., Ribeiro S.T., Tort A.B. (2011). Neuronal assembly detection and cell membership specification by principal component analysis. PLoS One.

[bb0145] Lopes-dos-Santos V., Ribeiro S., Tort A.B. (2013). Detecting cell assemblies in large neuronal populations. J. Neurosci. Methods.

[bb0155] Mallet L. (2008). Subthalamic nucleus stimulation in severe obsessive-compulsive disorder. N. Engl. J. Med..

[bb0160] Mallet N., Pogosyan A., Marton L.F., Bolam J.P., Brown P., Magill P.J. (2008). Parkinsonian beta oscillations in the external globus pallidus and their relationship with subthalamic nucleus activity. J. Neurosci..

[bb0165] Mallet N., Pogosyan A., Sharott A., Csicsvari J., Bolam J.P., Brown P., Magill P.J. (2008). Disrupted dopamine transmission and the emergence of exaggerated beta oscillations in subthalamic nucleus and cerebral cortex. J. Neurosci..

[bb0170] McNamara C.G., Rothwell M., Sharott A. (2022). Stable, interactive modulation of neuronal oscillations produced through brain-machine equilibrium. Cell Rep.

[bb0175] Moran A., Bar-Gad I. (2010). Revealing neuronal functional organization through the relation between multi-scale oscillatory extracellular signals. J. Neurosci. Methods.

[bb0180] Nakamura K.C., Sharott A., Tanaka T., Magill P.J. (2021). Input zone-selective dysrhythmia in motor thalamus after dopamine depletion. J. Neurosci..

[bb0185] Neumann W.J., Degen K., Schneider G.H., Brucke C., Huebl J., Brown P., Kuhn A.A. (2016). Subthalamic synchronized oscillatory activity correlates with motor impairment in patients with Parkinson’s disease. Mov. Disord..

[bb0190] Peters A.J., Fabre J.M.J., Steinmetz N.A., Harris K.D., Carandini M. (2021). Striatal activity topographically reflects cortical activity. Nature.

[bb0195] Peyrache A., Benchenane K., Khamassi M., Wiener S.I., Battaglia F.P. (2010). Principal component analysis of ensemble recordings reveals cell assemblies at high temporal resolution. J. Comput. Neurosci..

[bb0200] Raz A., Frechter-Mazar V., Feingold A., Abeles M., Vaadia E., Bergman H. (2001). Activity of pallidal and striatal tonically active neurons is correlated in mptp-treated monkeys but not in normal monkeys. J. Neurosci..

[bb0205] Sharott A., Magill P.J., Harnack D., Kupsch A., Meissner W., Brown P. (2005). Dopamine depletion increases the power and coherence of beta-oscillations in the cerebral cortex and subthalamic nucleus of the awake rat. Eur. J. Neurosci..

[bb0210] Sharott A., Gulberti A., Zittel S., Tudor Jones A.A., Fickel U., Munchau A., Koppen J.A., Gerloff C., Westphal M., Buhmann C., Hamel W., Engel A.K., Moll C.K. (2014). Activity parameters of subthalamic nucleus neurons selectively predict motor symptom severity in Parkinson’s disease. J. Neurosci..

[bb0215] Sharott A., Vinciati F., Nakamura K.C., Magill P.J. (2017). A population of indirect pathway striatal projection neurons is selectively entrained to parkinsonian Beta oscillations. J. Neurosci..

[bb0220] Steriade M. (2000). Corticothalamic resonance, states of vigilance and mentation. Neuroscience.

[bb0225] Tass P.A. (2003). A model of desynchronizing deep brain stimulation with a demand-controlled coordinated reset of neural subpopulations. Biol. Cybern..

[bb0230] Tass P.A., Qin L., Hauptmann C., Dovero S., Bezard E., Boraud T., Meissner W.G. (2012). Coordinated reset has sustained aftereffects in parkinsonian monkeys. Ann. Neurol..

[bb0235] Tinkhauser G., Pogosyan A., Tan H., Herz D.M., Kuhn A.A., Brown P. (2017). Beta burst dynamics in Parkinson’s disease OFF and ON dopaminergic medication. Brain.

[bb0240] Tinkhauser G., Pogosyan A., Little S., Beudel M., Herz D.M., Tan H., Brown P. (2017). The modulatory effect of adaptive deep brain stimulation on beta bursts in Parkinson’s disease. Brain.

[bb0245] Tremblay L., Filion M., Bedard P.J. (1989). Responses of pallidal neurons to striatal stimulation in monkeys with MPTP-induced parkinsonism. Brain Res..

[bb0250] Trouche S., Koren V., Doig N.M., Ellender T.J., El-Gaby M., Lopes-Dos-Santos V., Reeve H.M., Perestenko P.V., Garas F.N., Magill P.J., Sharott A., Dupret D. (2019). A Hippocampus-Accumbens Tripartite Neuronal Motif Guides Appetitive Memory in Space. Cell.

[bb0255] van de Ven G.M., Trouche S., McNamara C.G., Allen K., Dupret D. (2016). Hippocampal offline reactivation consolidates recently formed cell assembly patterns during sharp wave-ripples. Neuron.

