# Supplementary material for: Beta bursts in the parkinsonian cortico-basal ganglia network form spatially discrete ensembles
Source: Neurobiol Dis. 2024 Oct 15;201:106652. doi: 10.1016/j.nbd.2024.106652 (PMC11496931; doi:10.1016/j.nbd.2024.106652)
Supplement: Supplementary file 1 — Supplementary material [file mmc1.pdf]

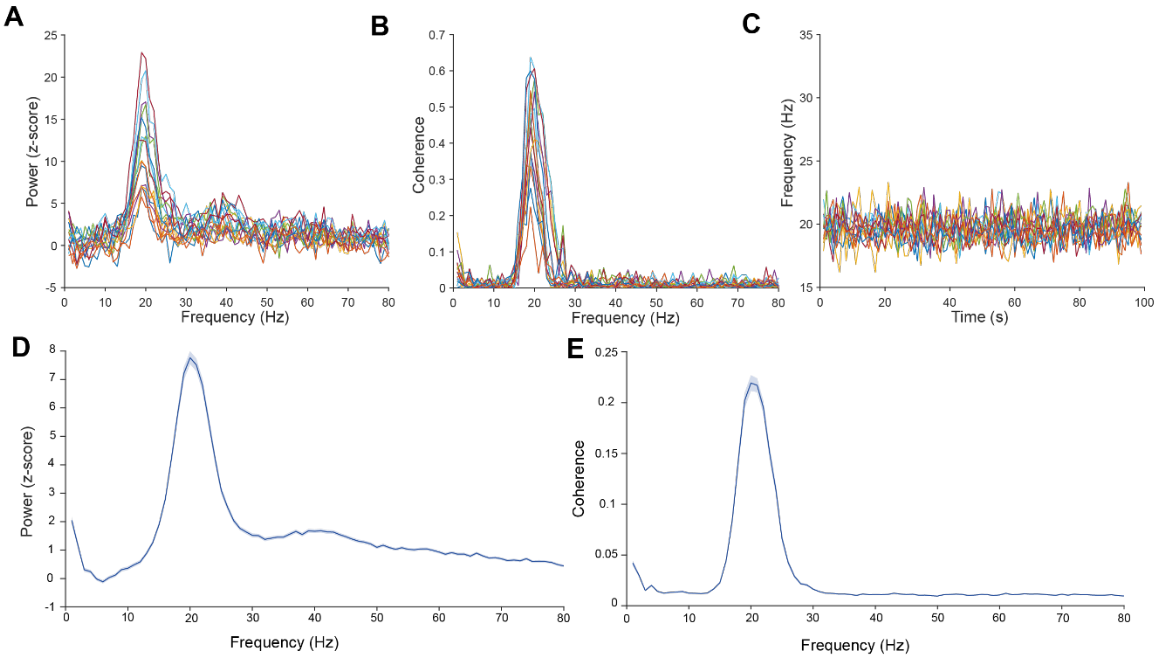

Figure S1, there is a strong, stable beta oscillation in the lesioned animals: **A**: The normalized power spectra of the BUA of 16 channels in the GP recorded in one session. The log power spectrum for each channel was z-scored relative to the mean and std of the high frequency (100-150Hz) log power spectrum. **B**: The coherence of the BUA of 16 channels in the GP recorded in one session with the ipsilateral ECoG. **C**: The frequency of beta-filtered BUA of 16 channels in the GP recorded in one session, calculated by the number of cycles over a second period. The frequency of oscillations was consistent across the duration of recordings. **D**: The mean normalized power spectra of the BUA of all channels in the GP. The log power spectrum for each channel was z-scored relative to the mean and std of the high frequency (100-150Hz) log power spectrum. **E**: The mean coherence of all of the recorded GP channels with the ipsilateral ECoG.

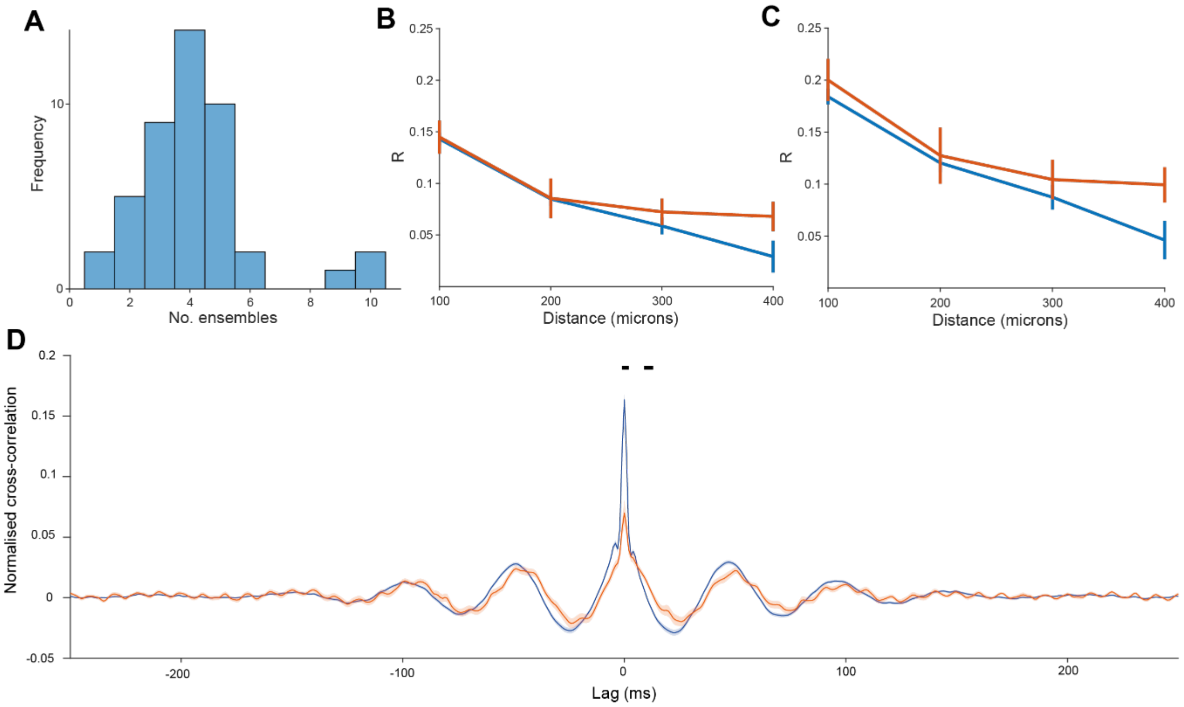

Figure S2, PCA-ICA applied to the raw-BUA did not replicate the properties of beta ensembles: Here we apply the PCA-ICA methodology to the raw-BUA. This yielded more ensembles than when we used the change in beta envelope. This is because there was more data for raw-BUA than for the change in beta-envelope. Raw-BUA was sampled at 1000Hz, whereas the change in envelope was calculated at 20Hz (over 50ms periods). There is therefore 50x more data for raw-BUA than the change in beta envelope, giving much more power for the identification of ensembles. However, the ensembles we do find do not replicate the beta-properties of beta ensembles. **A:** histogram showing the number of raw-BUA ensembles identified per recording. **B:** The average Pearson's R for the change in beta envelope was the same between pairs of member channels of raw-BUA ensembles (blue) and pairs of non-member channels (orange) over all distances (Wilcoxon rank sum test, p>0.05 for 100, 200, 300 and 400 microns)**. C:** The average Pearson's R for the beta envelope (undifferenced) was the same between pairs of member channels of raw-BUA ensembles (blue) and pairs of non-member channels (orange) over all distances (Wilcoxon rank sum test, p>0.05 for 100, 200, 300 and 400 microns)**. D:** The normalized cross correlation between pairs of member channels of raw-BUA ensembles (blue) and pairs of non-member channels (orange) at lags of -250ms to 250ms. Black markers above the graph show significance between the cross correlation of pairs of members and pairs of non-member channels. Significance was determined with the Wilcoxon rank sum test using false discovery rate statistics to control for the multiple time points compared. Unlike beta ensembles, significance was localised to only lags < 15ms (see **Fig 5** for comparison).

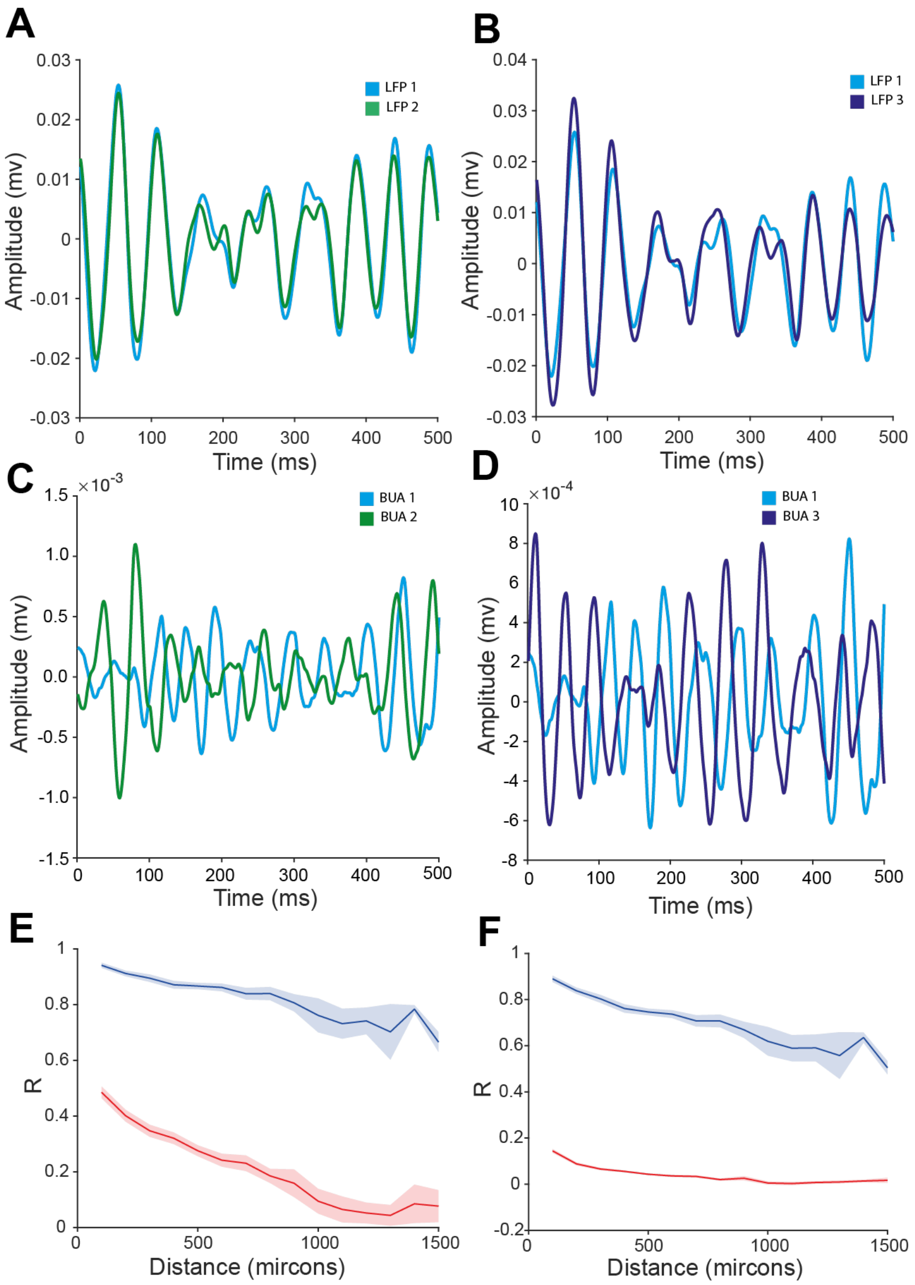

Figure S3, correlations in the change of the envelope of beta filtered BUA do not arise as a result of volume conduction: **A** and **B**: Exemplary beta filtered LFPs from 2 channels separated by 100 microns (LFP 1 and LFP 2 coloured in blue and green respectively) and 1000 microns respectively (LFP 1 and LFP 3 coloured in blue and purple respectively). **C** and **D**: Exemplary beta filtered BUAs from 2 channels separated by 100 microns (BUA 1 and BUA 2 coloured in blue and green respectively) and 1000 microns respectively (BUA 1 and BUA 3 coloured in blue and purple respectively). **E**: The average absolute Pearson's R of the beta-filtered LFP (blue) or beta-filtered BUA (red) between pairs of channels over variable distances. **F**: The average absolute Pearson's R of the change in beta-envelope over 50ms of the LFP (blue) or BUA (red) between pairs of channels over variable distances. The correlation between the beta-filtered signal and the change in the beta envelope over 50ms was much greater across pairs of LFPs than BUAs across all distances. Furthermore, whilst the correlation across all measures decreased with distance, this decrease was less steep for the LFP-derived signals than for BUAs.

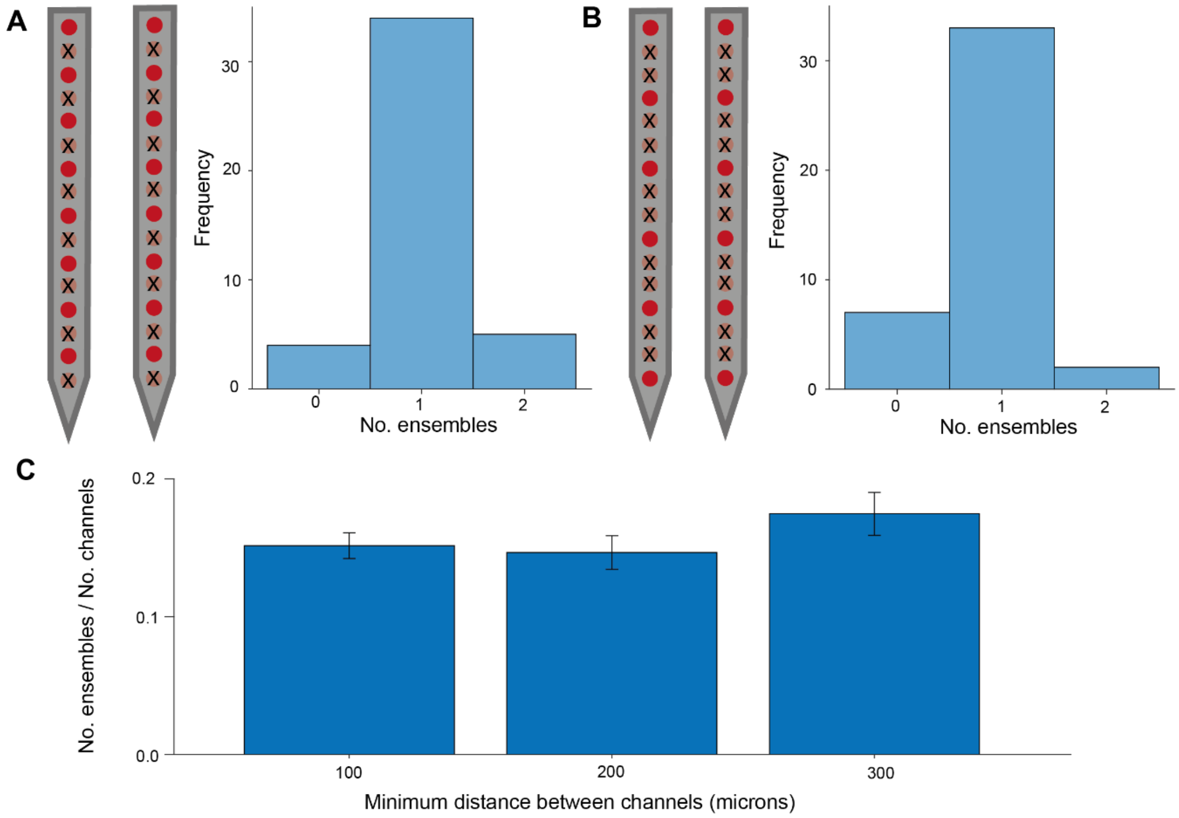

Figure S4, beta ensembles were detected even when channels were separated by larger distances: Here we exclude channels from the analysis to create a minimum spacing of 200 (**A**) and 300 (**B**) microns between channels. Excluding channels reduces the initial dimensionality of the data and thus reduces the number of beta ensembles that can be detected. However, importantly, ensembles can still be reliably detected even with greater distances between channels and the number of ensembles per channel was unchanged even as the distance between channels increased. **A:** a minimum distance between channels of 200 microns was created by excluding channels. **Left:** A schematic showing how channel exclusion would work on two probes each of which have 16 channels inside a target structure. Excluded channels are marked with a cross. **Right:** A histogram of the number of ensembles detected per recording. **B:** a minimum distance between channels of 300 microns was created by excluding channels. **Left:** A schematic showing how channel exclusion would work on two probes each of which have 16 channels inside a target structure. Excluded channels are marked with a cross. **Right:** A histogram of the number of ensembles detected per recording. **C:** There was no significant difference between the number of ensembles per channel as the minimum distance between channels increased (t-test for 100 vs 200 microns, 100 vs 300 microns and 200 vs 300 microns, $p>0.15$ for all).

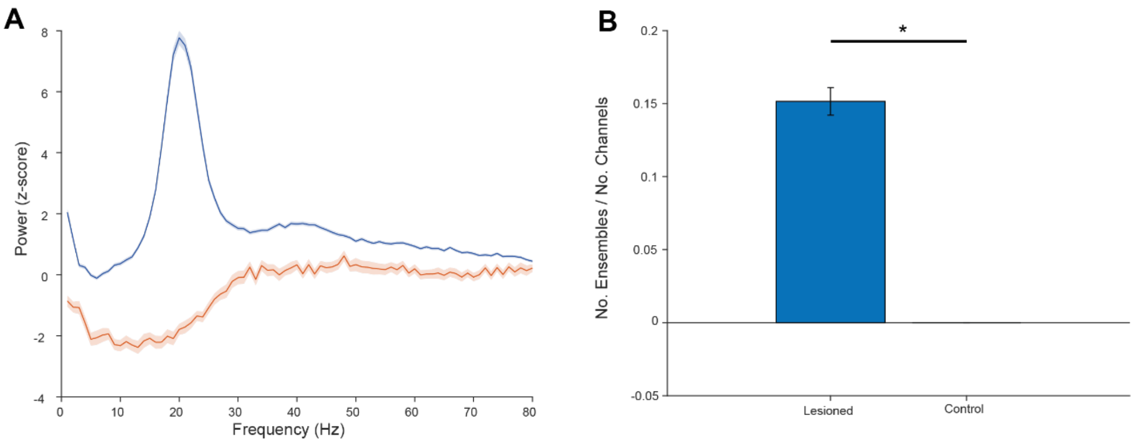

Figure S5, control rats had no peak in beta-power in BUA and beta ensembles could not be identified. Data from 5 control (no lesion) rats over 8 recording sessions was analyzed. **A:** The average log power spectrum of channels z-scored relative to the mean and std of the high frequency (100-150Hz) log power spectrum from the lesioned animals (blue) as compared with the control animals (orange). No beta-peak was observed in the control rats. **B:** The number of beta ensembles per channel in lesioned as compared to control animals. No beta ensembles were observed across the 8 recordings in the control animals. As a result, significantly more beta ensembles per channel were found in the lesioned animals as compared to the controls (t-test, $p<10^{-7}$).
